# DDGWizard: Integration of feature calculation resources for analysis and prediction of changes in protein thermostability upon point mutations

**DOI:** 10.1371/journal.pcbi.1013783

**Published:** 2025-12-01

**Authors:** Mingkai Wang, Khaled Jumah, Qun Shao, Katarzyna Kamieniecka, Yihan Liu, Krzysztof Poterlowicz

**Affiliations:** 1 Institute of Health and Social Care, University of Bradford, Bradford, United Kingdom; 2 Key Laboratory of Industrial Fermentation Microbiology, Ministry of Education, Tianjin Key Laboratory of Industrial Microbiology, The College of Biotechnology, Tianjin University of Science and Technology, Tianjin, China; Northeastern University, UNITED STATES OF AMERICA

## Abstract

Thermostability is an important property of proteins and a critical factor for their wide application. Accurate prediction of ΔΔG enables the estimation of the impact of mutations on thermostability in advance. A range of ΔΔG prediction methods based on machine learning has now emerged. However, their prediction performance remains limited due to insufficiently informative training features and little effort has been made to integrate feature calculation resources. Based on this, we integrated 12 computational resources to develop a pipeline capable of automatically calculating 1,547 features. In addition, a feature-enriched DDGWizard dataset was created, including 15,752 ΔΔG data. Furthermore, we performed feature selection and developed an accurate ΔΔG prediction model that achieved an *R*^2^ of 0.61 in cross-validation. It also outperformed several other representative prediction methods in comparisons with independent datasets. Together, the feature calculation pipeline, DDGWizard dataset, and prediction model constitute the DDGWizard system, freely available for ΔΔG analysis and prediction.

## Author summary

A protein’s ability to maintain its structure under high temperatures, known as thermostability, is critical for many industrial and therapeutic applications and might be affected by genetic mutations. To address the challenge, we built a robust machine learning model to predict the impact of mutations on thermostability. DDGWizard integrates data from multiple computational tools to calculate over 1,500 features for each mutation, offering detailed insights into protein structure and stability. DDGWizard simplifies the complex process of analysis and enables scientists to design more stable proteins for various applications. It bridges the gap between data-rich resources and practical tools. Our model demonstrated superior performance compared to existing methods and provides a freely accessible platform for researchers and industry professionals available at https://github.com/bioinfbrad/DDGWizard.

## Introduction

Thermostability is an important property of proteins, representing their ability to resist irreversible changes in structure and chemical attributes due to elevation in temperature [1]. It highly influences the application scope of proteins. For therapeutic proteins, such as monoclonal antibodies, insufficient thermostability can result in denaturation or reduced potency when temperature excursions occur during manufacturing, storage, and transportation [2], undermining their effectiveness. In addition, thermostability determines whether partial food proteins, such as whey proteins, can withstand thermal treatments [3], which is important in food processing to extend shelf life or create desired flavours [4]. For enzymes, specialized proteins widely used as biological catalysts, thermostability is a crucial parameter to function extensively [5]. As accelerating reactions, improving substrate solubility, and reducing the risk of microbial contamination require high temperatures in industrial environments, only enzymes with sufficient thermostability can operate continuously and be reused effectively [6]. However, most naturally evolved enzymes have poor thermostability [7], significantly limiting their applications.

Continuous efforts have been made to increase the thermostability of proteins [6] employing a variety of strategies. Directed evolution (DE) has been widely applied in protein engineering to increase protein thermostability [8–11]. It simulates natural selection and involves key steps such as constructing mutation libraries, introducing random mutations, and screening the target protein based on specific criteria. However, a major drawback of DE is its high demand for labor, material, and financial resources to identify the desired protein [12]. To identify effective mutations to increase protein thermostability more precisely, rational and semi-rational design strategies have been applied, which often require prior knowledge or computational methods [6]. ΔΔG is an indicator of protein thermostability changes resulting from mutations, as it represents the difference in the folding free energy change between the wild-type and mutant protein [13]. Since accurate ΔΔG prediction enables the estimation of the impacts of mutations on thermostability in advance, it can assist in the rational design of the selective introduction of mutations [14–16].

Early ΔΔG prediction methods are mainly based on empirical force fields [17], utilizing experimental parameters, classical equations, and energy evaluations to calculate ΔΔG, such as the classic FoldX prediction method [18]. With the continuous advancement of computational techniques and data science, ΔΔG prediction methods based on machine learning (ML) have emerged and are now widely adopted. Among the 23 ΔΔG prediction methods previously reviewed, 15 are based on ML [17]. However, despite their increase in number, current ML-based ΔΔG prediction methods still suffer from the issue of inadequate prediction performance [19–22]. One of the main reasons for this is that the features used for training models are insufficiently informative [19]. ACDC-NN [23] employs a neural network and optimizes for antisymmetric properties; however, its input features consist only of encodings of mutation type and amino acid distributions around the mutation site, lacking the integration of direct prior knowledge [24]. mCSM [25] and DynaMut [26] introduce pharmacophore features and protein dynamics features based on normal mode analysis (NMA), but they do not consider richer protein information, such as evolutionary conservation, residue interactions, and a broader range of amino acid physicochemical properties. DUET [27] relies solely on the prediction outputs of two other methods, SDM [28] and mCSM [25], as input features. In addition, some methods, such as DDGun3D [29] and FoldX [18], rely on linear fitting, which oversimplifies the problem and might be difficult to represent complex protein conformation changes. Finally, the size and protein diversity of some training datasets are limited [20], which may hinder model generalization (S1 Table lists the algorithms, datasets, and feature sets of ACDC-NN, DDGun3D, mCSM, DynaMut, FoldX, SDM, and DUET).

So far, although many computational resources have been used to calculate ΔΔG features [25,26,30,31] or output potentially relevant features [32–35], little effort has been made to integrate these resources for the comprehensive calculation of features for ΔΔG data. This could provide more diverse information, facilitating further analysis, feature selection, and ΔΔG prediction.

Here, we describe DDGWizard as a ΔΔG analysis system. It includes a feature calculation pipeline that integrates 12 computational resources [18,32–42] and is capable of automatically calculating 1,547 features for ΔΔG data. The calculated features provide information for the ΔΔG prediction from various perspectives, including the structure and environment of wild-type proteins, structural and environmental changes before and after mutation, mutation types, and evolutionary information. In addition, it provides a feature-enriched dataset created using the pipeline, including 15,752 ΔΔG data. Furthermore, it incorporates an accurate ΔΔG prediction model developed with the selected optimal features. The model achieved an *R*^2^ of 0.61 in cross-validation. It also outperformed several other prediction methods ACDC-NN [23], DDGun3D [29], FoldX [18], DynaMut [26], DUET [27], mCSM [25], and SDM [28]. The application program, datasets, and source code for DDGWizard training and validation have been published to ensure accessibility and reproducibility.

## Results

### An overview of DDGWizard

**Fig 1:**
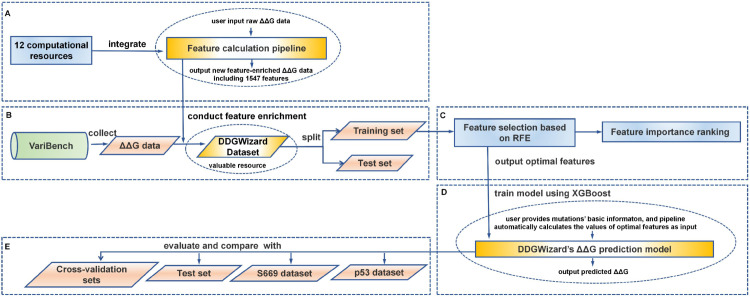
An overview of DDGWizard. A: Integrate 12 computational resources [18,32–42] to develop a feature calculation pipeline. B: Collect ΔΔG data from the VariBench [43] database, conduct feature enrichment to the collected ΔΔG data using the feature calculation pipeline to obtain the DDGWizard dataset, and then split it into training and test sets for subsequent ML tasks. C: Perform feature selection based on the RFE (recursive feature elimination) algorithm, followed by a further analysis of feature importance. D: Develop a ΔΔG prediction model using the XGBoost [44] algorithm based on the optimal features. E: Evaluate the developed model and compare it with other representative ΔΔG prediction methods using the identical cross-validation sets, test set, S669 dataset [45], and p53 dataset [25].

DDGWizard is a comprehensive ΔΔG analysis system. It incorporates a feature calculation pipeline, provides a feature-enriched dataset, and includes an accurate ΔΔG prediction model. The process of its development and validation includes five steps (as shown in Fig 1).

### DDGWizard feature calculation pipeline

**Fig 2:**
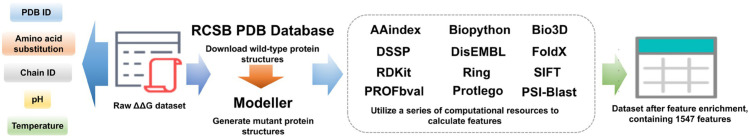
The feature calculation pipeline of DDGWizard. The pipeline requires the input of raw ΔΔG data (PDB ID, amino acid substitution, chain ID, pH, temperature, and ΔΔG value). It uses the PDB ID to download the wild-type protein structure file from the RCSB PDB database [46], employs Modeller [72] to construct the mutant protein structure file, and calls a series of computational resources [18,32–42] to calculate features, ultimately outputting the dataset containing 1,547 calculated features.

The ΔΔG feature calculation pipeline was developed by integrating 12 computational resources [18,32–42] (see Table 1) to obtain structural, environmental, and evolutionary information for proteins and associated mutation types. It requires raw ΔΔG data as input, including basic information on PDB ID [46] (e.g., 2OCJ for the p53 protein [25]), amino acid substitution (e.g., K6Q for lysine-to-glutamine at position 6), chain identifier (e.g., “A”), pH, temperature (in °C), and ΔΔG value. The computational resources are called to calculate the features, and users can then access the feature-enriched ΔΔG data, which totally includes 1,547 features (Fig 2).

**Table 1:** The computational resources used for feature calculation.

Computational Resources	Description	Contribution for Feature Calculation	Examples of ΔΔG Prediction Methods that Previously Used This or Similar Resource for Feature Calculation
AAIndex [36]	Database of amino acid physicochemical properties, substitution matrices and statistical protein contact potentials.	The physicochemical properties of amino acids and their changes are directly or indirectly related to the protein thermostability [47]. AAindex database provides various recorded values of physicochemical properties and substitution matrices for amino acids and it is used to output those as features.	PON-tstab [47], DDMut [48]
Biopython (v1.81) [37]	Python-based bioinformatics library.	The type of amino acids affects the distribution and strength of intramolecular interactions within a protein, thereby altering its structure and function [49]. Biopython is used to read protein sequence and structure files to calculate the proportions of different amino acids and different amino acid categories (uncharged polar, positively charged polar, negatively charged polar, nonpolar , aromatic, aliphatic, heterocyclic and sulfur-containing) of wild-type and mutant proteins as features.	PON-tstab, DDMut
Bio3D (v2.4) [38]	R package for structural bioinformatics.	The Bio3D library enables computationally inexpensive dynamics analysis of proteins based on NMA (Normal Mode Analysis) [50]. The atomic fluctuations analyzed by this method reflect the flexibility of protein regions, related to local structural stability [51]. The analysis of atomic fluctuations at mutation sites is used as features.	DynaMut [26]
DisEMBL (v1.5) [32]	Tool for intrinsic protein disorder prediction.	Disordered regions in proteins often lack stable tertiary structures, making them the initial sites of thermal unfolding and thereby reducing the protein’s thermostability [52]. DisEMBL is used to predict the disordered region distribution of both wild-type and mutant proteins as features.	-
DSSP [39]	Programme that determines the secondary structure of proteins	Both secondary structure types and the solvent-accessible surface area of residues are important factors influencing local protein stability [53,54]. DSSP is used to calculate the secondary structure distribution and RSA (relative solvent accessibility) of amino acids for both the wild-type and mutant proteins.	DDGun [29], PremPS [55], NeEMO [56]
FoldX (v5.0) [18]	Protein structure analysis tools based on empirical force fields.	The empirical energy terms output by FoldX have been widely used [57–59] to estimate Gibbs free energy changes and analyze protein stability. The 20 energy terms [18] calculated by FoldX for both the wild-type and mutant proteins are used as features.	STRUM [31], ELASPIC [30], Prethermut [60]
RDkit [40]	Cheminformatics software.	Pharmacophore features can identify the active sites of proteins [61], which often serve as “trade-off regions" between stability and function [62]. RDKit is used to calculate the pharmacophore distribution in both wild-type and mutant proteins as features.	mCSM [25], DDMut
PROFbval [34]	Protein B-factor (temperature factor) prediction tool.	The B-factor (temperature factor) can assess the flexibility of residues [63], which is associated with local structural stability. PROFbval is used to predict the B-factors of wild-type and mutant amino acids.	-
Protlego (v1.81) [35]	Protein design and analysis tools.	Protlego can compute the distribution of hydrophobic clusters within proteins, which serve as a major driving force for protein folding and play a critical role in maintaining stability [64,65]. The calculated distribution of hydrophobic clusters in the wild-type and mutant protein structures is used as features.	-
PSI-BLAST (v2.13.0+) [41]	Protein sequence alignment and homology search tools.	PSI-BLAST can perform multiple sequence alignment against sequences in the database to generate a PSSM (Position-Specific Scoring Matrix), which estimates the probability of each amino acid occurring at each position, thereby reflecting the evolutionary conservation of that site [66]. Changes in conservation caused by mutations often have a significant impact on thermostability [67]. PSSM scores surrounding the mutation site for both the wild-type and mutant proteins are used as features.	DeepDDG [68], STRUM, PROTS-RF [69]
Ring (v3.0) [33]	Residue interaction network generator.	The structure and function of a protein rely heavily on its internal interactions [70]. Ring is used to calculate the distribution of different types of interactions (hydrogen bonds, disulfide bridges, ionic interactions, Van der Waals forces, π−cation, and π−π stacking) within the wild-type and mutant proteins.	NeEMO
SIFT [42]	Tool for predicting effects of amino acid substitutions on proteins	SIFT can predict whether a protein functionally tolerates a given mutation [42] and functional intolerance to mutations is often associated with structural or stability perturbations [71]. The results of SIFT prediction are used as features to reflect the mutation type.	ELASPIC, STRUM

The description of the calculated features and the corresponding computational resources is provided below.

**Structural and environmental information of the wild-type protein.** The first feature group incorporates structural information within the wild-type protein, covering the proportion of different amino acids and different amino acid categories (uncharged polar, positively charged polar, negatively charged polar, nonpolar, aromatic, aliphatic, heterocyclic and sulfur-containing) calculated with Biopython [37], buried/exposed amino acids and different secondary structures ($3_{10}$-helix, alpha-helix, pi-helix, helix-turn, extended beta sheet, beta bridge, bend and other/loop) obtained from DSSP [39], disordered regions predicted by DisEMBL [32], different residue interactions (hydrogen bonds, disulfide bridges, ionic interactions, Van der Waals forces, π−cation, and *π* − *π* stacking) output by Ring [33], different atomic pharmacophores [25] (hydrophobic, positive, negative, hydrogen acceptor, hydrogen donor, aromatic, sulphur, and neutral) calculated with RDKit [40], and hydrophobic clusters analyzed by Protlego [35]. To account for the varying effects of residues and protein conformations at different distances from the mutation site, structural information is divided into four spatial regions: within 7 Å of the mutation site, within 10 Å of the mutation site, within 13 Å of the mutation site, and across the entire protein structure.

Subsequently, different properties of wild-type amino acids are included, including RSA (Relative Solvent Accessibility) calculated by DSSP [39], atomic fluctuation information based on NMA (Normal Mode Analysis) [50] by Bio3D [38], B-factor (Temperature Factor) predicted by Profbval [34], and the physicochemical properties recorded in the AAindex database [36].

Finally, the energy information of the wild-type protein is incorporated from FoldX [18]. In total, the first group includes 724 features.

**Structural and environmental changes between mutant and wild-type proteins.** The second group contains 647 features to describe the changes in structure and environment between mutant and wild-type proteins. First, the features are calculated for the mutant protein in the similar manner as it has been done for the wild protein using the computational resources described above. Subsequently, the difference in the feature values between the mutant and wild-type proteins constitutes this feature group. Considering that some features of the structural proportion of proteins do not show significant changes before and after single-point mutations, such as the proportion of disordered regions and buried/exposed amino acids, these features have not been included.

**Types of mutations.** The third group includes 146 features to describe the mutation types. Various encodings are incorporated to represent information on amino acid substitutions, such as substitution encoding for changes of amino acid types, secondary structures, and residue interactions on the mutated amino acids. Subsequently, values from amino acid substitution matrices in the AAindex database [36] are also included to describe mutation types. Finally, the tool SIFT [42]’s prediction results, to reflect the impact of amino acid substitutions on proteins, are also encoded to represent the mutation types.

**Evolutionary information.** The fourth group includes 26 features to describe the evolutionary information. These features are statistics from the PSSM (position-specific scoring matrix) generated by the protein sequence alignment and homology search tools PSI-BLAST [41]. The PSSM scores at the mutation site and surrounding sites of both the wild-type and mutant proteins are included. Additionally, the difference in PSSM scores at the mutation site between the mutant and wild-type proteins, and the difference in the average PSSM scores surrounding the mutation site between the mutant and wild-type proteins, are also included.

### Feature-enriched DDGWizard dataset

**Fig 3:**
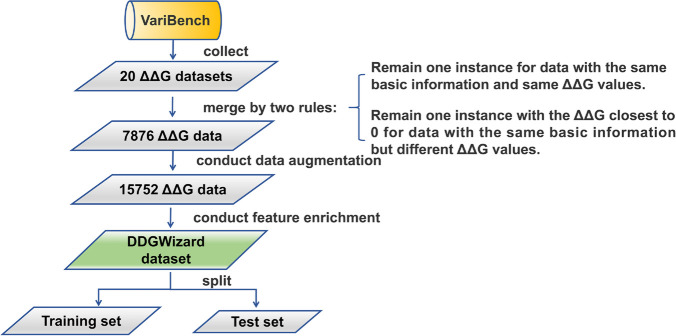
The workflow of dataset construction and feature enrichment.

Fig 3 demonstrates the workflow of dataset construction and feature enrichment. We chose the VariBench database [43] as the data source. VariBench is a database that curates previously validated mutation datasets, including ΔΔG datasets. A total of 20 raw ΔΔG datasets were collected (see S2 Table) that met the requirements of including five pieces of basic mutation information (PDB ID, amino acid substitution, chain identifier, pH and temperature) and experimental ΔΔG values. To maximize data utility, we merged these 20 datasets based on the following merging rules:

- For data with the same basic information and the same ΔΔG value, we retained only one instance.
- For data with the same basic information but different ΔΔG values, we selected one instance with the ΔΔG value closest to 0 (according to the previous report [73], current ΔΔG data have a trend toward to 0, the ΔΔG data closer to 0 could be more reliable).

After merging, we obtained 7,876 unique ΔΔG mutation data points from 222 different proteins. Considering that the hypothetical reverse mutation theory has been adopted by many ΔΔG studies [19,23,29,74], both in the testing [45,75,76] and development [77–79] of ΔΔG prediction methods, we conducted the data augmentation that added the hypothetical reverse mutations, eventually obtaining 15,752 ΔΔG data.

**Fig 4:**
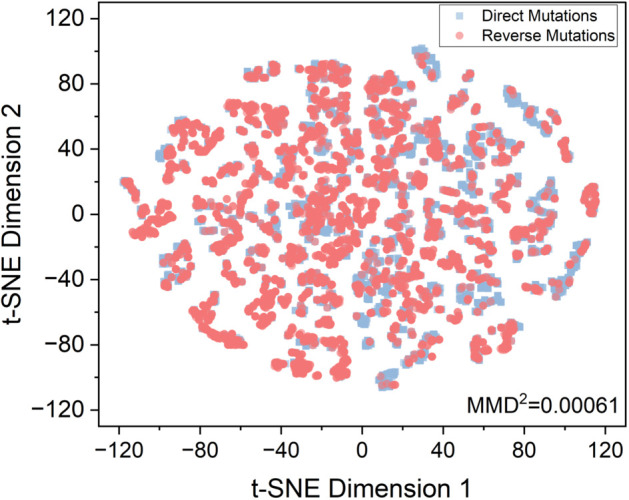
t-SNE plot for both direct and reverse mutation data. The t-SNE plot shows the distribution of direct and reverse mutation data, projected from high-dimensional feature spaces into two dimensions. The blue points represent direct mutation data, while the red points represent reverse mutation data. *MMD*^2^ quantifies the difference in feature distributions between the two types of data.

We applied the developed feature calculation pipeline to the obtained ΔΔG data. It enriched the feature number of the data from 5 to 1,547. Fig 4 shows the distribution of feature-enriched data and highlights the similarity of direct and reverse mutation data with an *MMD*^2^ [80] of 0.0006. It reflects that the reverse mutation data could approximately serve as an equivalent augmentation of the dataset [81].

The created new dataset was named “DDGWizard” dataset. It is a non-redundant collection including unique 15,752 mutation ΔΔG data points from 222 proteins and integrated comprehensive feature information covering measuring conditions, structures and environments of the wild-type protein, structural and environmental changes between mutant and wild-type proteins, mutation types, and evolutionary information, making it a valuable resource for feature selection, development of ML models, and further ΔΔG analytical studies.

Next, we split the dataset for ML tasks. Each pair of direct mutation data and hypothetical reverse mutation data in the DDGWizard dataset was treated as a single unit, and all pairs were randomly shuffled using a seed of 42. The first 90% data was selected as the training set, comprising 14,178 ΔΔG mutations (7,089 pairs of direct and reverse mutations) from 219 different proteins. The remaining 10% data was selected as the test set, comprising 1,594 ΔΔG mutations (787 pairs of direct and reverse mutations) from 134 different proteins.

### Optimal ΔΔG feature set

**Fig 5:**
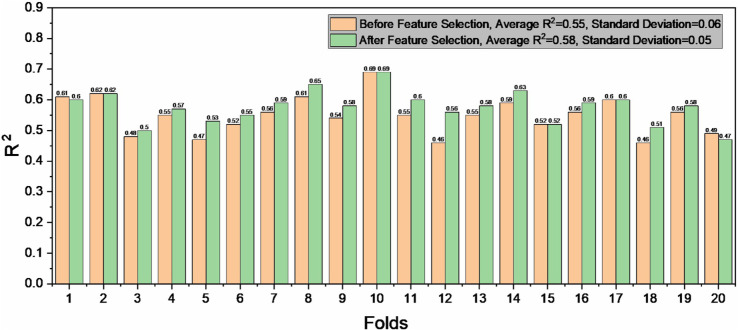
*R*^2^ of each fold from cross-validation before and after feature selection.

To identify the most effective features, feature selection was carried out. We first trained the model with the XGBoost algorithm [44] using all 1,574 features as baseline. The 20-fold pair-level cross-validation (it ensures that the direct and reverse mutation data remain together in either the training set or the validation set) was used to evaluate the model training performance. Fig 5 shows the performance of the model before feature selection, with an average *R*^2^ of 0.55 and a standard deviation of 0.06.

**Fig 6:**
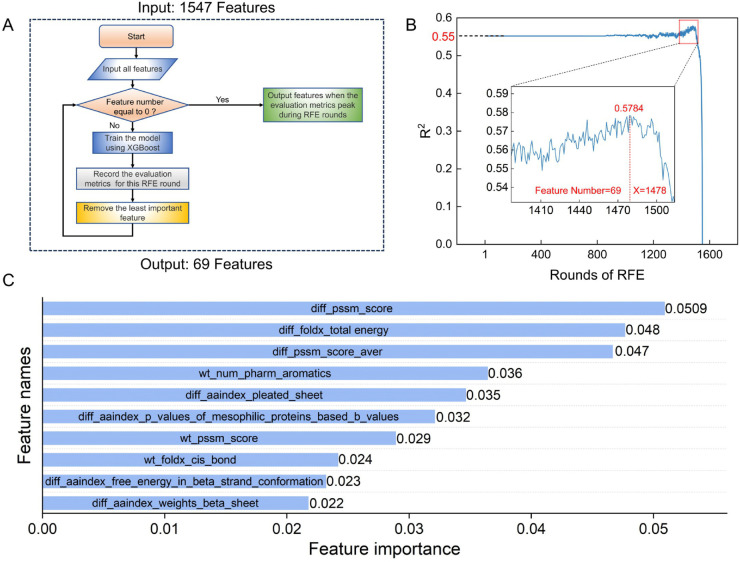
Feature selection and feature importance ranking. A: The flowchart of feature selection based on the RFE algorithm. B: The RFE results reflect the changes in the average R2 of the 20-fold pair-level cross-validation as the number of RFE rounds increases and the number of features decreases. C: The top 10 most important features among the 69 features.

Next, the RFE algorithm was employed to select features, which iteratively removes the least important features and outputs the evaluation metric in each round (the flowchart of RFE is shown in Fig 6A). Fig 6B shows the changes in average *R*^2^ across the RFE rounds. The RFE curve performed relatively stable or showed few fluctuations during the elimination of the first 1,397 features. When features were reduced to fewer than 150, the prediction performance began to improve. When RFE reached 1,478 rounds, reducing the features to 69, prediction performance peaked with an average *R*^2^ of 0.58. Fig 5 compares the model’s performance before and after feature selection. The average *R*^2^ increased by 0.03 when the model was trained with the selected 69 features. In addition, the standard deviation of *R*^2^ decreased from 0.06 to 0.05.

The optimal 69 features are listed in S3 Table, including evolutionary features, energy terms, changes in amino acid physicochemical properties, RSA (relative solvent accessibility) at the mutation site, temperature, and distributions of amino acid categories, secondary structures, residue interactions, atomic pharmacophores, disorder regioins, and hydrophobic clusters. These features were used for further analysis and model development.

Moreover, we used the XGBoost algorithm to output the feature importance (the top 10 most important features are shown in Table 2 and Fig 6C, respectively). The most important feature is “diff_pssm_score”, which represents the difference in PSSM scores at the mutation site between the mutant and wild-type proteins. In addition, two other evolutionary features, “diff_pssm_score_aver” (the change in the average PSSM value of the surrounding sequence at the mutation site), and “wt_PSSM_score” (the PSSM value at the mutation site in the wild-type protein) are also among the top 10 important features. Since the PSSM score provides a quantitative measure of the conservation degree of amino acids at a specific site [82], the difference in the PSSM scores between mutant and wild-type amino acids reflects how well the mutation aligns with the preferred amino acid at the site. Larger differences indicate a greater deviation from the most favorable amino acid at that position. Such deviations may affect the function or structure of the protein, as conservation at these positions often suggests that they are essential to maintain its integrity [83]. The second most important feature is “diff_foldx_total_energy”, which represents the difference in the overall energy, calculated by FoldX [18], between mutant and wild-type proteins. It shows that empirical force field methods like FoldX can effectively assist ML methods for ΔΔG predictions. It is worth mentioning that the four features, reflecting changes in physicochemical properties derived from the AAindex, are ranked among the top 10 features. Among them, the feature “diff_aaindex_p_values_of_mesophilic_proteins_based_b_values” can reflect the statistical significance changes in protein thermostability for mesophilic proteins based on the distributions of b values [84]; the other three features reflect changes in parameters associated with different secondary structures at the mutation site [85–87].

**Table 2:** Details of the 10 most important features.

Feature Names	Feature Description	Feature Importance
diff_PSSM_score	The difference in PSSM scores at the mutation site between the mutant and wild-type proteins.	0.051
diff_foldx_total energy	The difference in overall energy calculated by FoldX between mutant and wild-type proteins.	0.048
diff_PSSM_score_aver	The difference in the average PSSM scores surrounding the mutation site between the mutant and wild-type proteins.	0.047
wt_num_pharm_c_aromatics	The number of aromatic pharmacophores in the wild-type protein.	0.036
diff_aaindex_pleated_sheet	The difference in the information measure for pleated sheet between mutant and wild-type amino acids.	0.035
diff_aaindex_p_values_of_mesophilic_proteins_based_b_values	The difference in the p-values of mesophilic proteins based on the distribution of b-values between mutant and wild-type amino acids.	0.032
wt_PSSM_score	The PSSM value of the wild-type protein at the mutation site.	0.029
wt_foldx_cis_bond	The energy information of the cis peptide bond of wild-type protein.	0.024
diff_aaindex_free energy_in_beta_strand_ conformation	The difference in the free energy in the beta strand conformation between mutant and wild-type amino acids.	0.023
diff_aaindex_weights_beta_sheet	The difference in the weights for beta-sheet between mutant and wild-type amino acids.	0.022

### Model development and evaluation

The XGBoost algorithm was chosen to train the ΔΔG prediction model of DDGWizard. Table 3 presents the results of a model selection, comparing the performance of 11 machine learning (ML) algorithms: AdaBoost [88], decision tree [89], KNN [90], Lasso regression [91], LightGBM [92], linear regression [93], MLP [94], random forest [95], Gaussian process [96], support vector regression [97], and XGBoost [44]. Traditional ML algorithms were evaluated with their default hyperparameters, while the tuning of MLP hyperparameters is summarized in S4 Table. Among these algorithms, XGBoost achieved the highest average *R*^2^ of 0.55 under the same 20-fold pair-level cross-validation.

**Table 3:** Average *R*^2^ for the model selection under the same 20-fold pair-level cross-validation.

Algorithm	Average *R*^2^	Standard deviation of *R*^2^
XGBoost [44]	0.55	0.06
LightGBM [92]	0.53	0.05
Random Forest [95]	0.53	0.06
MLP [94]	0.42	0.07
Gaussian Process [96]	0.34	0.09
Liner Regression [93]	0.33	0.06
KNN [90]	0.30	0.07
Lasso Regression [91]	0.26	0.05
Support Vector Regression [97]	0.24	0.09
AdaBoost [88]	0.17	0.04
Decision Tree [89]	0.12	0.14

We then trained the model using the optimal 69 features with the XGBoost algorithm. Bayesian optimization [98] was employed to tune the model’s hyperparameters, with the average *R*^2^ during the 20-fold pair-level cross-validation as the optimization target (specific parameter ranges and tuning results can be found in S5 Table). After Bayesian optimization, the average *R*^2^ of the model training improved from 0.58 to 0.61.

**Fig 7:**
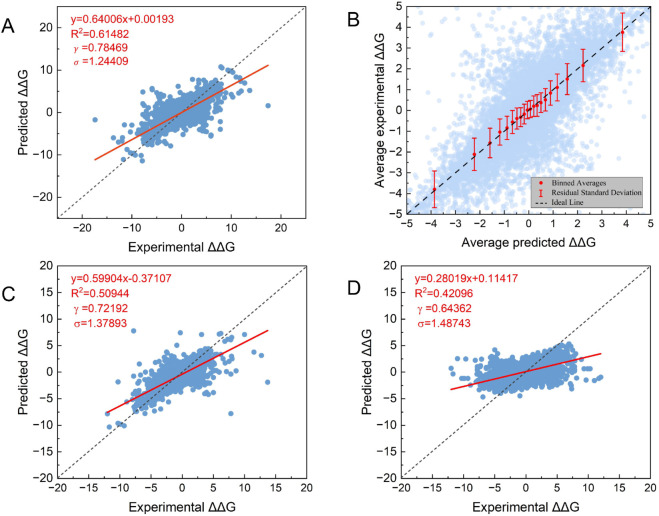
Prediction results of DDGWizard’s model from the cross-validation. A: The scatter plot to visualize the comparison between all predicted and true values. The red line indicates the overall regression fit. The plot also provides the regression equation, R2, Î³, and Ïƒ values for the overall prediction. B: The binned scatter plot compares average prediction values and experimental values within 10 bins that have equivalent data amounts. The error bars represent the standard error of the residuals between the average predicted and true values within each bin. C: The scatter plot to visualize the prediction results from the 20-fold cross-validation on the 3,970 mutation Î"Î"G data points where the PSSM score of the mutant amino acid is less than 0. D: The scatter plot to visualize the prediction results from the 20-fold protein-level crossvalidation on the 30 proteins that have mutual sequence similarity less than 30%.

Fig 7A shows the prediction results during cross-validation, while Fig 7B demonstrates the comparison between the average ΔΔG prediction values and experimental values within 10 bins that have equivalent data amount [99–101]. The distribution of 10 comparison points around y=x indicates model’s good calibration and strong reliability.

To assess the robustness of our model on low-conservation residue data, we conducted 20-fold cross-validation using data where the PSSM score of the mutant amino acid was less than 0 (a total of 3,970 data points), representing relatively low conservation of the mutant amino acid [82]. Fig 7C shows the test results, and our model achieved an average *R*^2^ of 0.51 under the same optimal features and hyperparameters as used before.

To test our model’s performance on proteins with low mutual sequence similarity (<30%), we selected 30 proteins (PDB IDs: 1BNI, 1W3D, 1VQB, 1STN, 3SSI, 1RX4, 2LZM, 1RTB, 1LZ1, 2CI2, 1FKJ, 1DIV, 2ABD, 1UZC, 3MBP, 1FTG, 1RN1, 1ARR, 1TEN, 1AMQ, 2RN2, 1YYJ, 1APS, 5PTI, 1HZ6, 1SAK, 1OTR, 1PIN, 5AZU, 1TTG) with at least 50 mutations in our dataset. We then performed 20-fold protein-level cross-validation [31]. As shown in Fig. 7D, our model achieved an average *R*^2^ of 0.42.

To evaluate the impact of inclusion of reverse mutation data on model performance, we conducted a comparison study (Table 4). We first performed 20-fold cross-validation with direct mutation data for both training and validation dataset, which yielded an average *R*^2^ of 0.58 (Table 4, row 1). Next, we added the corresponding reverse mutation data into the training sets while keeping the validation sets unchanged, and the average *R*^2^ remained 0.58 (Table 4, row 2). This indicates that adding reverse mutation data to the training set does not significantly affect the prediction performance on direct mutations under different data splits. In the third experiment, we used direct and reverse mutation data as both training and validation sets and a 20-fold pair-level cross-validation was conducted, which obtained an average *R*^2^ of 0.61 (Table 4, row 3). The final experiment included direct mutation data for the training set, and direct and reverse mutation data for the validation sets, and the average *R*^2^ dropped to 0.26 (Table 4, row 4). It suggests that including reverse mutation data in the training set can effectively improve the prediction performance on reverse mutations and therefore enhance models’ generalization ability.

**Table 4:** Comparison study on the inclusion of reverse mutation data.

Training sets	Validation sets	Average *R*^2^	Std of *R*^2^
Direct mutations only	Direct mutations only	0.58	0.06
Direct mutations + Reverse mutations	Direct mutations only	0.58	0.06
Direct mutations + Reverse mutations	Direct mutations + Reverse mutations	0.61	0.05
Direct mutations only	Direct mutations + Reverse mutations	0.26	0.04

The model with the highest *R*^2^ (0.73) on the validation set from the 20-fold pair-level cross-validation was selected as DDGWizard’s ΔΔG prediction model. For new ΔΔG prediction needs, users need to provide basic mutation information on PDB ID, amino acid substitution, chain identifier, pH, and temperature, and the developed feature calculation pipeline will automatically calculate the optimal 69 feature values to input into the prediction model. The model will then output the predicted ΔΔG values.

### Comparisons

To compare the performance differences between DDGWizard’s ΔΔG prediction model and others, seven representative methods were chosen for the comparison, including ACDC-NN [23], DDGun3D [29], FoldX [18], DynaMut [26], DUET [27], mCSM [25], and SDM [28]. S1 Table provides information on the algorithms, datasets, and feature sets used by these methods. We conducted four comparisons using different datasets: identical cross-validation sets, test set, S669 dataset [45] and p53 dataset [25]. All test datasets have undergone data augmentation, enabling evaluation of the prediction methods’ performance in predicting all data, direct mutation data, and reverse mutation data.

#### Comparison with the cross-validation sets

**Fig 8:**
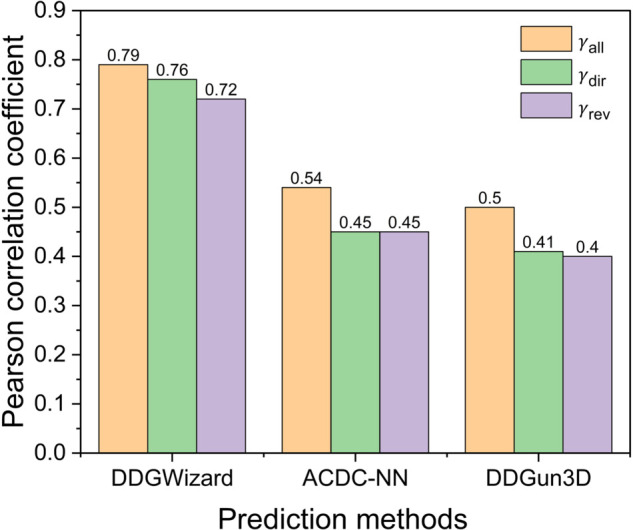
Pearson correlation coefficients of three ΔΔG prediction methods evaluated with the identical cross-validation sets.

To initially compare DDGWizard’s ΔΔG prediction model with other prediction methods, we first selected two representative prediction methods to compare: ACDC-NN [23] and DDGun3D [29]. These two methods were ranked as the top two methods in the previous study [45]. We used ACDC-NN and DDGun3D to predict identical pair-level cross-validation sets that DDGWizard used and compared their prediction performance with the DDGWizard’s model. Table 5 and Fig 8 present the comparison results, showing that DDGWizard’s model significantly outperforms ACDC-NN and DDGun3D, achieving $\gamma_{all}$, $\gamma_{dir}$, and $\gamma_{rev}$ values of 0.79, 0.76, and 0.72 ($\gamma_{all}$, $\gamma_{dir}$, and $\gamma_{rev}$ represent the Pearson correlation coefficient between the predicted and true values for all data, direct mutation data, and reverse mutation data, respectively). Statistical significance was confirmed by *z*_*all*_ and *p*_*all*_ (significance metrics for correlation coefficient comparison derived from Steiger’s Z-test [102,103]), with *z*_*all*_ exceeding 50 and *p*_*all*_ less than 0.001. All three prediction methods were constructed with consideration of the hypothetical reverse mutation theory, and the effectiveness of this consideration was reflected in the models’ antisymmetric property [23]. The values of $\gamma_{dir,rev}$ (Pearson correlation coefficient between the predicted values of direct mutation data and reverse mutation data) for the three methods are close to the ideal prediction of –1, and the values of δ (the average of the sums of the predicted values for each pair of direct and reverse mutation data) are similarly close to the ideal prediction of 0.

**Table 5:** Comparison results of three ΔΔG prediction methods evaluated with the identical cross-validation sets.

Methods	$\gamma_{\text{all }}$	$z_{all}$	$p_{all}$	$\sigma_{\text{all }}$	$\gamma_{\text{dir }}$	$\sigma_{\text{dir }}$	$\gamma_{\text{rev }}$	$\sigma_{\text{rev }}$	$\gamma_{\text{dir,rev }}$	δ
DDGWizard	0.79	–	–	1.24	0.76	1.20	0.72	1.29	–0.95	0
ACDC-NN	0.54	50.80	<0.001	1.70	0.45	1.70	0.45	1.70	–1	0
DDGun3D	0.50	56.17	<0.001	1.77	0.41	1.77	0.40	1.76	–0.98	–0.03

We also compared the three prediction methods using the identical cross-validation sets on the low-conservation residue data and low similarity proteins. The DDGWizard’s model achieved better performance than ACDC-NN and DDGun3D with $\gamma_{all}$ of 0.64 and 0.72 (see S6 Table and S7 Table), respectively.

#### Comparison with the test set

**Fig 9:**
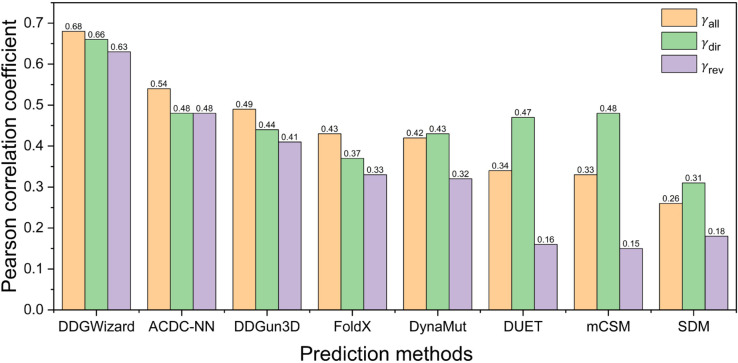
Pearson correlation coefficients of eight ΔΔG prediction methods evaluated with the test set.

To further compare performance differences between the DDGWizard’s ΔΔG prediction model and other ΔΔG prediction methods, we selected additional five representative methods which are FoldX [18], DynaMut [26], DUET [27], mCSM [25], and SDM [28] to predict the test set. Table 6 and Fig 9 present the test results of eight ΔΔG prediction methods. As shown, the DDGWizard’s model achieved the best prediction performance when predicting all data (with a $\gamma_{all}$ of 0.68), direct mutation data (with a $\gamma_{dir}$ of 0.66), and reverse mutation (with a $\gamma_{rev}$ of 0.63). Its performance advantage is also statistically significant, as all *p*_*all*_ from comparisons with other methods were less than 0.001. In terms of the comparison of antisymmetric property [23], DDGWizard’s model, ACDC-NN, and DDGun3D significantly outperformed other methods.

**Table 6:** Comparison results of eight ΔΔG prediction methods evaluated with the test set.

Methods	$\gamma_{\text{all }}$	$z_{all}$	$p_{all}$	$\sigma_{\text{all }}$	$\gamma_{\text{dir }}$	$\sigma_{\text{dir }}$	$\gamma_{\text{rev }}$	$\sigma_{\text{rev }}$	$\gamma_{\text{dir,rev }}$	δ
DDGWizard	0.68	–	–	1.48	0.66	1.46	0.63	1.50	–0.96	0
ACDC-NN	0.54	7.69	<0.001	1.69	0.48	1.69	0.48	1.69	–1	0
DDGun3D	0.49	10	<0.001	1.76	0.44	1.75	0.41	1.77	–0.98	–0.03
FoldX	0.43	12.44	<0.001	2.10	0.37	2.10	0.33	2.11	–0.75	–0.10
DynaMut	0.42	12.10	<0.001	1.82	0.43	1.75	0.32	1.89	–0.65	–0.10
DUET	0.34	14.96	<0.001	2.01	0.47	1.70	0.16	2.29	–0.34	–0.64
mCSM	0.33	17.23	<0.001	2.07	0.48	1.69	0.15	2.39	–0.27	–0.83
SDM	0.26	14.78	<0.001	2.12	0.31	1.97	0.18	2.25	–0.60	–0.39

#### Comparison with the S669 dataset

**Fig 10:**
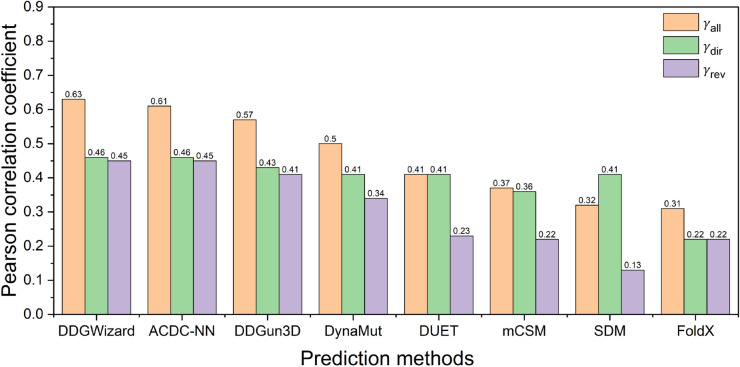
Pearson correlation coefficients of eight ΔΔG prediction methods evaluated with the dataset S669.

Table 7 and Fig 10 present the test results on the widely used [48,104,105] S699 dataset [45] for the eight prediction methods, including the DDGWizard’s model. Since 43 mutation data points from S669 were included in our training set, we excluded these data and retrained [77–79] DDGWizard’s model using the same features and hyperparameters as before for comparison. In the evaluation on S669, the DDGWizard’s model, ACDC-NN, and DDGun3D remained the top-performing ΔΔG prediction methods. Our model achieved the highest $\gamma_{all}$ of 0.63, and ACDC-NN exhibited the best anti-symmetric performance with $\gamma_{dir,rev}$ of –0.98.

**Table 7:** Comparison results of eight ΔΔG prediction methods evaluated with the dataset S669.

Methods	$\gamma_{\text{all }}$	$z_{all}$	$p_{all}$	$\sigma_{\text{all }}$	$\gamma_{\text{dir }}$	$\sigma_{\text{dir }}$	$\gamma_{\text{rev }}$	$\sigma_{\text{rev }}$	$\gamma_{\text{dir,rev }}$	δ
DDGWizard	0.63	–	–	1.58	0.47	1.6	0.44	1.54	–0.92	0.09
ACDC-NN	0.61	0.92	0.17	1.5	0.46	1.49	0.45	1.5	–0.98	–0.02
DDGun3D	0.57	4.44	<0.001	1.61	0.43	1.6	0.41	1.62	–0.97	–0.05
DynaMut	0.5	14.04	<0.001	1.65	0.41	1.6	0.34	1.69	–0.58	–0.06
DUET	0.41	7.19	<0.001	1.86	0.41	1.52	0.23	2.14	–0.12	–0.67
mCSM	0.37	11.46	<0.001	1.96	0.36	1.54	0.22	2.3	–0.05	–0.85
SDM	0.32	12.31	<0.001	1.93	0.41	1.67	0.13	2.16	–0.4	–0.4
FoldX	0.31	10.01	<0.001	2.39	0.22	2.3	0.22	2.48	–0.2	–0.34

#### Comparison with the p53 dataset

**Fig 11:**
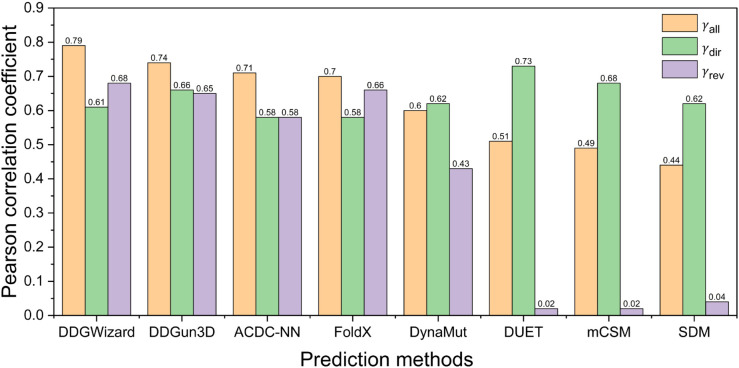
Pearson correlation coefficients of eight ΔΔG prediction methods evaluated with the p53 dataset.

Table 8 and Fig 11 present the test results on the p53 dataset [25] for the eight ΔΔG prediction methods, including the DDGWizard’s model. As four data from the dataset p53 were included in DDGWizard’s training data, we excluded these data and retrained [23,31,56] DDGWizard’s model using the same features and hyperparameters as before for comparison. Based on the ranking of $\gamma_{all}$, DDGWizard’s model outperformed the other methods (0.79).

**Table 8:** Comparison results of eight ΔΔG prediction methods evaluated with the p53 dataset.

Methods	$\gamma_{\text{all }}$	$z_{all}$	$p_{all}$	$\sigma_{\text{all }}$	$\gamma_{\text{dir }}$	$\sigma_{\text{dir }}$	$\gamma_{\text{rev }}$	$\sigma_{\text{rev }}$	$\gamma_{\text{dir,rev }}$	δ
DDGWizard	0.79	–	–	1.52	0.61	1.58	0.68	1.46	–0.89	0.06
DDGun3D	0.74	0.97	0.16	1.59	0.66	1.56	0.65	1.61	–1.02	–0.03
ACDC-NN	0.71	1.83	0.03	1.72	0.58	1.72	0.58	1.72	–1.02	0
FoldX	0.70	1.67	0.04	1.95	0.58	1.95	0.66	1.94	–0.56	0.43
DynaMut	0.60	2.95	<0.001	1.90	0.62	1.72	0.43	2.07	–0.54	–0.16
DUET	0.51	3.97	<0.001	2.13	0.73	1.31	0.02	2.71	–0.16	–0.79
mCSM	0.49	4.55	<0.001	2.22	0.68	1.40	0.02	2.81	–0.06	–0.91
SDM	0.44	3.86	<0.001	2.11	0.62	1.54	0.04	2.56	–0.54	–0.45

### Accessibility and reproducibility

We developed DDGWizard as a freely available system for ΔΔG analysis and prediction. The user can access the DDGWizard application on https://github.com/bioinfbrad/DDGWizard. The feature calculation pipeline requires to input raw ΔΔG data and outputs new data with 1,574 features. The DDGWizard’s ΔΔG prediction model requires to provide basic mutation information and it returns predicted ΔΔG values. Both of feature calculation pipeline and ΔΔG prediction model support parallel processing to handle large-scale data. To better assist users in predicting ΔΔG, the program also provides tools for ΔΔG prediction of saturation mutagenesis and full-site mutagenesis. Detailed usage instructions can be found at https://ddgwizard.readthedocs.io/en/latest/. The DDGWizard dataset, the source code for model training and validation, and the evaluation and comparison data are released on https://zenodo.org/records/14512134.

## Discussion

Thermostability has a significant impact on the broad applications of proteins. Continuous efforts have been made to increase protein thermostability, employing various strategies, such as rational design or semi-rational design. Since ΔΔG prediction can estimate the impact of mutations on thermostability in advance, it has become a powerful tool for rational or semi-rational design. Although a range of ΔΔG prediction methods have been developed, especially those based on ML, they still suffer from inadequate prediction performance. The main reason for this is that the features used for training models are insufficiently informative. In fact, many computational resources are available to calculate the features for ΔΔG predictions. However, there is a lack of work to integrate these resources for comprehensive calculation. It could provide more diverse feature information, facilitating further analysis, feature selection, and ΔΔG prediction.

In this study, we integrated 12 computational resources [18,32–42] to develop a pipeline to aid users in feature enrichment for their own ΔΔG datasets. It can automatically output 1,547 calculated features, covering diverse information, such as the structures and environments of wild-type proteins, structural and environmental changes between mutant and wild-type proteins, mutation types, and evolutionary information. Furthermore, we collected ΔΔG data and applied our pipeline to create the feature-enriched DDGWizard dataset, including 15,752 data points, serving as a valuable resource for ΔΔG research.

In addition, to identify more effective features for ΔΔG prediction, we carried out feature selection based on RFE (recursive feature elimination). During this process, the RFE curve first remained stable over a long range and then began to rise. At the peak, 69 features were selected as the optimal subset, resulting in a more accurate and robust model with improved *R*^2^ and a decreased standard. This can be attributed to the elimination of redundant features, allowing the model to focus on more informative ones [106]. Similar RFE patterns can be observed in previous studies [107–110]. According to importance ranking of optimal features, we found that the difference in PSSM scores at the mutation site between mutant and wild-type proteins was the most important feature. This may be because changes in the PSSM score at the mutation site can reflect how well the mutation matches the preferred amino acid at that position. Larger differences indicate greater deviation, which may potentially affect the protein’s function or structure, since conserved positions are often critical for maintaining integrity. Besides, we found that the energy terms derived from FoldX and changes in physicochemical properties related to certain secondary structures are also important for prediction.

Finally, using the optimal features, we developed an accurate new ΔΔG prediction model. It outperformed ACDC-NN [23], DDGun3D [29], FoldX [18], DynaMut [26], DUET [27], mCSM [25], and SDM [28]. ACDC-NN employs a convolutional neural network and optimizes for antisymmetric properties. However, its input features include only encodings of mutation type and amino acid distribution around the mutation site, lacking the utilization of prior knowledge–based features [24]. This limits the model’s interpretability and may increase the risk of overfitting [111]. In contrast, the features used in our model have more direct contributions to ΔΔG due to knowledge-based feature design. Moreover, the training set it uses, S2648 dataset [112] (also employed by DynaMut [26], mCSM [25], and DUET [27]), contains 132 source proteins that are entirely covered by the 219 proteins in our training set. As a result, it has been trained on a relatively narrower range of proteins than our model, which could limit its generalization performance. DDGun3D uses four features to represent differences in conservation, hydrophobicity, sequence interaction energy, and structural interaction energy between mutant and wild-type amino acids, and it fits ΔΔG values through a linear combination. While this approach is intuitive, the linear combination may be insufficient to capture the complex nonlinear relationships between features and ΔΔG. A similar limitation is observed in FoldX [18], which computes rich and complex conformational energy terms of proteins but only performs simple linear weighting of these terms. mCSM and DynaMut introduce pharmacophore features and protein dynamics features based on NMA (normal mode analysis) [50], using Gaussian processes and random forest algorithms, respectively, to train their models. However, both methods don’t train with amino acid conservation features [66,82] that are important features found in our results. In addition, they did not incorporate the XGBoost algorithm, which demonstrated better performance in our model selection than the algorithms adopted in their models. Furthermore, they do not consider hypothetical reverse mutations [19], which hinders their models to learn reverse mutations’ patterns, resulting in relatively low $\gamma_{dir,rev}$ (Pearson correlation between predictions of direct and reverse mutations). SDM [28] is a statistical potential function based on an environment-specific amino acid substitution table. While statistical approaches are valuable for understanding data distributions, their reliance on prior assumptions about data distributions might lead to prediction biases on new data [113]. DUET [27] is a consensus predictor that uses the outputs of SDM and mCSM as features and applies the SVM for training. Across the comparisons, DUET’s performance is slightly better than SDM and mCSM individually, indicating the effectiveness of consensus prediction. However, its accuracy is still significantly lower than that of the top-performing methods.

Our current work focuses on integrating features from 12 computational resources [18,32–42] based on expert knowledge, identifying the optimal subset from 1,574 integrated features using RFE, and developing an accurate ΔΔG prediction model with XGBoost. While XGBoost is a powerful tool that is effective for structured data and provides strong interpretability [114], its limitation lies in that it cannot perform complex transformations of input features to automatically learn new feature representations and contextual patterns in the data [115–117]. We aim to address this problem in our future work. We will explore the incorporation of deep learning (DL) to further improve model accuracy. DL allows the automated extraction of abstract representations [118] from data and often achieves better performance on large-scale datasets, despite its limited interpretability. DL-based representation of sequence conservation, such as the output embeddings from pre-trained protein language models (PLMs) [119], could be introduced. DL algorithms GNN [120] or CNN [121] could be utilized to further extract deep-learned representation from the distribution of amino acids, secondary structures, and amino acid interactions. We aim to integrate the current RFE-selected features with deep-learned representations to develop hybrid models for further improving model performance.

Overall, the ΔΔG analysis and prediction system, DDGWizard, consists of an integrated feature calculation pipeline, a feature-enriched dataset, and an accurate prediction model. The system is freely available, and the source code for its training and validation procedures has been published to ensure accessibility and reproducibility.

## Materials and methods

### Development of feature calculation pipeline

The feature calculation pipeline was developed in the Python programming language (v3.10.12). It was programmed to read raw ΔΔG data (PDB ID, amino acid substitution, chain identifier, pH, and temperature) as input. Then it downloads the structural files of the wild-type proteins from the RCSB PDB database [46] according to the provided PDB ID using the requests (v2.31.0) library and utilize the homology modeling software Modeller (v10.4) [72] to generate the mutant protein structures using the wild-type protein structure as template. Next, a series of computational resources [18,32–42] is called to calculate the feature values, and it finally saves the calculated results in CSV format. Detailed descriptions of the usage of each computational resource in the pipeline are provided in S8 Table.

### Data sources

In this study, three data sources were used:

**VariBench.** VariBench [43] is a benchmark database that includes mutation datasets, such as ΔΔG datasets, and follows seven principles (relevance; representative-ness; non-redundancy; experimentally verified cases; positive and negative cases; scalability; reusability) to improve the quality of the collected datasets. 20 datasets from the VariBench database were selected, which were further merged, filtered, and split to achieve the training set and test set used for ML tasks.

**S669.** The dataset S669 [45] contains 669 mutation data points from 87 different proteins. It is a high-quality benchmark dataset and has been used by several ΔΔG studies [77–79] for independent tests.

**p53.** The dataset p53 [25] contains 42 ΔΔG data of tumor suppressor proteins (PDB ID: 2OCJ). Since the p53 dataset is widely used for comparing and testing ΔΔG prediction methods [27,28,77,79], it was also adopted in this study for testing and comparison purposes.

### Data augmentation based on hypothetical reverse mutation theory

The changes in thermostability (ΔΔG) caused by protein mutations are represented by the difference in protein folding free energy (ΔG) between mutant and wild-type proteins. As a thermodynamic state function [122], the difference in ΔG should be reversible. Namely, at the same position in the protein, the ${\Delta \Delta G}_{A->B}$ for a mutation from amino acid A to amino acid B should be equal to the negative of the ${\Delta \Delta G}_{B->A}$ for the hypothetical reverse mutation from amino acid B to amino acid A [19] (as shown in Eq 1). This is known as the hypothetical reverse mutation theory.

$$\Delta \Delta G_{A\to \;B}=-\Delta \Delta G_{B\to \;A}$$ (1)

This theory has been widely applied in many ΔΔG studies [19,23,29,74], both in the testing [45,75,76] and development [77–79] of ΔΔG prediction methods. According to this theory, a robust ΔΔG prediction method should perform well not only in predicting direct mutations but also in predicting hypothetical reverse mutations [19]. In the test set, hypothetical reverse mutation data can be generated from each direct mutation data. This type of data augmentation for the test set allows comprehensive evaluations for prediction methods by additionally predicting reverse mutation data. In addition to being used in testing, this theory should also be applied in the construction of ΔΔG prediction methods. Previous studies [45] have shown that incorporating this theory can effectively improve methods’ prediction performance when predicting hypothetical reverse mutation data and allow methods to learn the antisymmetric property [23] of ΔΔG. In contrast, ΔΔG prediction methods that did not consider this theory achieved much poorer performance [45,75,76]. For ΔΔG prediction methods based on ML, the hypothetical reverse mutation theory can be incorporated to generate reverse mutation data in the training set for data augmentation [74,77–79].

### Pair-level cross-validation

Among the ΔΔG prediction methods [74,77–79] that utilized the hypothetical reverse mutation theory to increase data in the training set, mutation-level cross-validation (randomly shuffle all mutation data during cross-validation [31]) was employed by them. However, considering that a pair of real data and its hypothetical reverse mutation data are correlated, if they are randomly shuffled during cross-validation, one real data instance and its augmented data instance might be located in the training and validation sets, respectively. This could result in the validation set not being entirely unseen for the training set, leading to the training set and validation set not being independently separated. Previous study [123] suggested that, when conducting cross-validation after data augmentation, if training and validation data are not independently separated, data leakage might occur and overly optimistic performances could be caused. To address this issue, we employed the pair-level cross-validation, which means splitting datasets based on a pair of real data and its augmented data as a unit in the cross-validation. This ensures that each data pair appears entirely in the training set or in the validation set, preventing the potential issue of unfair validation.

### Feature selection

Feature selection is implemented using the RFE (Recursive Feature Elimination) algorithm. RFE can effectively eliminate redundant features and identify the optimal feature subset to improve model prediction performance, making it a widely used technique in various ML tasks [124–126]. RFE is an algorithm that relies on feature importance, and its basic idea is to iteratively train the model, evaluate the prediction performance of the model, calculate feature importance and remove the least important feature in each round, ultimately selecting a subset of features that contribute the most to the model’s prediction performance. In this study, RFE was implemented based on the RFECV function [127] from sklearn.feature_selection library [128]. The ML algorithm XGBoost was used to train the models during RFE rounds and output feature importance. The average *R*^2^ of 20-fold pair-level cross-validation was employed as the metric to evaluate the model performance for each RFE round. To be more specific, RFE performed the following three iterative steps (denoting the feature set at each round as X, which is initially set to include all candidate features):

- Train the XGBoost model using the feature set X, perform cross-validation, calculate the average *R*^2^, and record the result.
- Use the feature importance output by the XGBoost model to rank the features in descending order. Remove the lowest-ranked feature from X and record the remaining features.
- Repeat the step 1 and step 2 until all features have been removed from X.

After completing RFE, the remaining features corresponding to the round with the highest average *R*^2^ are selected as the optimal features, finalizing the feature selection process.

### Model development

The ΔΔG prediction model of DDGWizard was developed using the XGBoost [44] algorithm. The XGBoost algorithm is a powerful ML method [44] based on gradient boosting trees. It incorporates both the L1 and L2 regularization penalty terms to control the model complexity and reduce overfitting [129], while its post-split pruning strategy [130] further prevents unnecessary tree growth. The inclusion of L1 regularization also enables a more reliable estimation of feature importance [131], making it well-suited for integration with RFE-based feature selection. The implementation of XGBoost was achieved using the ML library scikit-learn (v1.3.1). The model’s hyperparameters were determined through Bayesian optimization [98], which is a sequential design strategy for global optimization of black-box functions, suitable for hyperparameter tuning in ML models. In this study, Bayesian optimization set the average *R*^2^ from the 20-fold pair-level cross-validation on dataset S7089 as the optimization target and was implemented using the library Bayesian optimization (v1.4.3).

### Evaluation metrics

MMD (maximum mean discrepancy) test [80] was conducted to evaluate the feature distribution difference between the direct and reverse mutation data. It is a widely used [132–134] method that quantifies the difference between two probability distributions in the high-dimensional space. The metrics *MMD*^2^ [80,135] was employed, and its formula is given by Eq 2 (where P and Q represent two distributions; samples x and y are drawn from distributions P and Q, with sizes m and n; k represents the RBF kernel function [80] implemented via the pairwise_kernels function of the sklearn.metrics library [128]):

$${MMD}^{2}(P,Q)=\frac{1}{m^{2}}\sum_{i=1}^{m}\sum_{j=1}^{m}k(x_{i},x_{j})+\frac{1}{n^{2}}\sum_{i=1}^{n}\sum_{j=1}^{n}k(y_{i},y_{j})-\frac{2}{mn}\sum_{i=1}^{m}\sum_{j=1}^{n}k(x_{i},y_{j})$$ (2)

During cross-validation for feature selection and model development, the coefficient of determination (*R*^2^) between real ΔΔG and predicted ΔΔG is used as the evaluation metric. Its formula is given by Eq 3 (where n is the total amount of data; $x_{i}$ and $y_{i}$ represent the predicted and real values for the number i data; $\overset{¯}{y}$ represents the mean of the real values):

$$R^{2}=1-\frac{\sum_{i=1}^{n}{\left(x_{i}-y_{i}\right)}^{2}}{\sum_{i=1}^{n}{\left(y_{i}-\overset{―}{y}\right)}^{2}}$$ (3)

In comparisons of ΔΔG prediction methods, a total of eight evaluation metrics, which were used in previous studies [23,45,75,79], were employed:

- Pearson correlation coefficient between the predicted and true values for all data, direct mutation data, and reverse mutation data (use $\gamma_{all}$, $\gamma_{dir}$, and $\gamma_{rev}$ to represent them, respectively).
- Root mean square error between predicted and true values for all data, direct mutation data, and reverse mutation data (use $\sigma_{all}$, $\sigma_{dir}$, and $\sigma_{rev}$ to represent them, respectively).
- Pearson correlation coefficient between the predicted values of the direct mutation data and reverse mutation data (use $\gamma_{dir,rev}$ to represent).
- The average of the sums of the predicted values for each pair of direct and reverse mutation data (use δ to represent).

The formula for calculating the Pearson correlation coefficient (*γ*) is given by Eq 4 (where n is the total amount of data; $x_{i}$ and $y_{i}$ represent the predicted and real values for the i-th data; $\overset{¯}{x}$ and $\overset{¯}{y}$ represent the means of the predicted and real values):

$$\gamma =\frac{\sum_{i=1}^{n}\left(x_{i}-\bar{x}\right)\left(y_{i}-y\right)}{\sqrt{\sum_{i=1}^{n}{\left(x_{i}-\overset{―}{x}\right)}^{2}\sum_{i=1}^{n}{\left(y_{i}-\overset{―}{y}\right)}^{2}}}$$ (4)

The formula for calculating the root mean square error (*σ*) is given by Eq 5 (where n is the total amount of data; $x_{i}$ and $y_{i}$ represent the predicted and real values for the number i data):

$$\sigma =\sqrt{\frac{1}{n}\sum_{i=1}^{n}{\left(x_{i}-y_{i}\right)}^{2}}$$ (5)

The formula for calculating the Pearson correlation coefficient between the predicted values of the direct mutation data and the reverse mutation data ($\gamma_{dir,rev}$) is given by Eq 6 (where n is the total number of pairs of the direct and reverse mutation data; $\Delta \Delta G_{i,dir}$ and $\Delta \Delta G_{i,rev}$ represent the predicted ΔΔG values for the i-th pair of the direct and reverse mutation data, respectively; $\overset{―}{\Delta \Delta G_{dir}}$ and $\overset{―}{\Delta \Delta G_{rev}}$ represent the means of all predicted ΔΔG values for the direct mutation data and reverse mutation data):

$$\gamma_{dir,rev}=\frac{\sum_{i=1}^{n}\left(\Delta \Delta G_{i,dir}-\overset{―}{\Delta \Delta G_{dir}}\right)\left(\Delta \Delta G_{i,rev}-\overset{―}{\Delta \Delta G_{rev}}\right)}{\sqrt{\sum_{i=1}^{n}{\left(\Delta \Delta G_{i,dir}-\overset{―}{\Delta \Delta G_{dir}}\right)}^{2}\sum_{i=1}^{n}{\left(\Delta \Delta G_{i,rev}-\overset{―}{\Delta \Delta G_{rev}}\right)}^{2}}}$$ (6)

The formula for calculating the average of the sums of the predicted values for each pair of direct and reverse mutation data (*δ*) is given by Eq 7 (where n is the total number of pairs of direct and reverse mutations; $\Delta \Delta G_{i,dir}$ and $\Delta \Delta G_{i,rev}$ respectively represent the predicted ΔΔG values for the i-th pair of direct and reverse mutation data):

$$\delta =\frac{\sum_{i=1}^{n}\left(\Delta \Delta G_{i,dir}+\Delta \Delta G_{i,rev}\right)}{n}$$ (7)

The $\gamma_{all}$ is the metric to rank compared methods. Steiger’s Z-test [102] was employed to evaluate the statistical significance of the differences in $\gamma_{all}$ between the DDGWizard’s model and other methods. It is a method for determining whether two correlation coefficients associated with the same target variable are statistically significantly different [136]. The test was implemented using the online server of Cocor [103]. The inputs included the $\gamma_{all}$ of the DDGWizard’s model, $\gamma_{all}$ of the compared methods, Pearson correlation coefficient between the predicted values of the DDGWizard’s model and the compared methods, and data number in the test set. The output included a Z-score (*z*_*all*_) and a p-value (*p*_*all*_). The *z*_*all*_ quantifies the statistical significance of the difference in $\gamma_{all}$ between the DDGWizard’s model and the compared methods, where a larger absolute value means stronger difference significance. The *p*_*all*_ (ranging from 0 to 1) represents the probability of obtaining the current statistical result or more extreme results under the null hypothesis [137] that there is no difference in $\gamma_{all}$ between the DDGWizard’s model and the compared methods.

## Supporting information

S1 TableList of algorithms, training datasets and feature sets used in representative ΔΔG prediction methods.(PDF)

S2 TableList of collected datasets.The list of 20 ΔΔG datasets that were collected from the VariBench database and merged.(PDF)

S3 Table List of the remaining 69 features from feature selection based on the RFE algorithm. (PDF)

S4 Table Performance comparison of different MLP hyperparamers with identical 20-fold pair-level cross-validation. (PDF)

S5 Table Used hyperparameters for Bayesian optimization. Hyperparameter ranges used for 100 rounds of Bayesian optimization.
(PDF)

S6 TableComparison results of three ΔΔG prediction methods evaluated with the identical cross-validation sets on the low-conservation residue data.(PDF)

S7 TableComparison results of three ΔΔG prediction methods evaluated with the identical protein-level cross-validation sets.(PDF)

S8 Table Detailed usage of computational resources. (PDF)
