# Supplementary material for: DDGWizard: Integration of feature calculation resources for analysis and prediction of changes in protein thermostability upon point mutations
Source: PLoS Comput Biol. 2025 Dec 1;21(12):e1013783. doi: 10.1371/journal.pcbi.1013783 (PMC12688154; doi:10.1371/journal.pcbi.1013783)
Supplement: S1 Table — (PDF) [file pcbi.1013783.s001.pdf]

S1 Table . List of algorithms, training datasets and feature sets used in representative ΔΔG prediction methods.

Methods	Algorithm	Traning dataset	Feature set
ACDC-NN [23]	Convolutional neural network	S2648 dataset[111], including 2,648 mutation data points from 132 proteins	20 features to encode the mutation amino acid, 100 features to encode the wild-type amino acid and its ±2 neighboring amino acids, and 500 features to encode the amino acids located within a 5 Å spatial radius around the mutation site.
DDGun3D [29]	Linear combination	PON-tstab dataset [47], including 1,564 mutation data points from 99 proteins (DDGun3D used this dataset to find linear fitting weights)	Four features to represent the differences in conservation, hydrophobicity, sequence interaction energy, and structural interaction energy between mutant and wild-type amino acids.
mCSM [25]	Gaussian process	S2648 dataset, including 2,648 mutation data points from 132 proteins	Graph representation of atomic pharmacophore distribution, pharmacophore changes, pH and temperature.
DynaMut [26]	Random forest	S2648 dataset, including 2,648 mutation data points from 132 proteins	NMA-based protein dynamic features [50], DUET prediction output, RSA (relative solvent accessibility) , residue depth, and secondary structure.
FoldX [18]	Linear combination	339 mutation data points from 9 proteins	Energy terms including entropy contribution, Van der Walls forces, hydrogen bonds, electrostatic interaction, etc.
SDM [28]	Statistical potential function based on an environment -specific amino acid substitution table	-	-
DUET [27]	Support vector machine	S2648 dataset, including 2,648 mutation data points from 132 proteins	SDM prediction output and mCSM prediction output.
