# Supplementary material for: DDGWizard: Integration of feature calculation resources for analysis and prediction of changes in protein thermostability upon point mutations
Source: PLoS Comput Biol. 2025 Dec 1;21(12):e1013783. doi: 10.1371/journal.pcbi.1013783 (PMC12688154; doi:10.1371/journal.pcbi.1013783)
Supplement: S2 Table — The list of 20 ΔΔG datasets that were collected from the VariBench database and merged. (PDF) [file pcbi.1013783.s002.pdf]

S2 Table . List of collected datasets. The list of 20 ΔΔG datasets that were collected from VariBench database and merged.

Dataset Names	Data Number	Data Number after Merging
Broom_S605	605	551
PoPMuSiC_S2648	2,648	1,435
EASE-MM_S1676	1,676	1,156
Saraboji_S2204	2,204	1,009
STRUM_Q3421	3,421	872
HotMuSiC_S1626	1,626	863
EASE-MM_S543	543	395
STRUM_Q306	306	128
iPTREE-STAB_S1859	1,859	498
M47 and M8_S2760	2,760	120
bty342	342	304
PON_Tstab	1,565	58
M47 and M8_S1810	1,810	91
D24_M3131	3,131	17
EASE-MM_S238	238	177
iStable2_S3568	3,568	111
I_Mutant2.0_S1948	1,948	1
iStable2_S630	630	40
Saraboji_S1396	1,396	28
SVM-3D12_S1634	1,634	22
