# Supplementary material for: DDGWizard: Integration of feature calculation resources for analysis and prediction of changes in protein thermostability upon point mutations
Source: PLoS Comput Biol. 2025 Dec 1;21(12):e1013783. doi: 10.1371/journal.pcbi.1013783 (PMC12688154; doi:10.1371/journal.pcbi.1013783)
Supplement: S3 Table — (PDF) [file pcbi.1013783.s003.pdf]

S3 Table . List of the remaining 69 features from feature selection based on the RFE algorithm (part 1).

Feature Names	Feature Importance
diff_PSSM_score	0.050909806
diff_foldx_total energy	0.047666822
diff_PSSM_score_aver	0.046654977
wt_num_pharm_c_aromatics	0.036433689
diff_aaindex_pleated_sheet	0.034640647
diff_aaindex_p_values_of_mesophilic_proteins_based_b_values	0.032110024
wt_PSSM_score	0.028912913
wt_foldx_cis_bond	0.024188913
diff_aaindex_free energy_in_beta_strand_ conformation	0.023194175
diff_aaindex_weights_beta_sheet	0.021750489
wt_num_PIPISTACK_ring	0.020166533
diff_foldx_solvation_hydrophobic	0.020024816
diff_aaindex_thermodynamic_beta_sheet_propensity	0.01919879
wt_foldx_disulfide	0.018727105
diff_aaindex_normalized_flexibility_parameters	0.01860607
diff_num_pharm_c_hb_acceptors	0.018154806
wt_pct_ss_-	0.015804831
wt_num_pharm_c_hydrophobics	0.015098524
wt_max_hd_cluster_area	0.014572973
mut_PSSM_score	0.014425991
wt_num_HBOND_ring	0.013867605
diff_num_pharm_c_hydrophobics	0.013433069
diff_aaindex_relative_population_of_conformational_state_E	0.013327676
diff_aaindex_side_chain_interaction_parameter	0.013133734
diff_num_pharm_c_hb_donors	0.012755573
wt_num_VDW_ring	0.012244292
diff_aaindex_mean_area_buried_on_transfer	0.012059962
layer3_wt_num_pharm_c_hydrophobics	0.012017773
wt_pct_ss_H	0.01175021
wt_RSA	0.011557183
wt_pct_ss_E	0.011099557
diff_aaindex_transfer_free_energy_from_chx_to_oct	0.011056727
wt_num_PIPISTACK_ring_layer2	0.011042171
diff_aaindex_normalized_positional_residue_frequency	0.010903099
diff_aaindex_average_relative_fractional_occurrence	0.010871695
diff_aaindex_hydration_potential	0.010714913
diff_aaindex_hydropathy_scale	0.010445237
diff_aaindex_average_number_of_surrounding_residues	0.010220053
layer2_wt_num_pharm_c_hb_acceptors	0.010176585
wt_aaindex_normalized_frequency_C_terminal_beta_sheet	0.01011178

S3 Table. List of the remaining 69 features from feature selection based on the RFE algorithm (part 2).

Feature Names	Feature Importance
diff_aaindex_normalized_frequency_turn_in_all_alpha_class	0.01004812
diff_aaindex_unfolding_Gibbs_energy_in_water	0.010031626
wt_foldx_helix_dipole	0.009842365
wt_pct_ss_T	0.009828604
layer_wt_num_pharm_c_hydrophobics	0.009787734
wt_foldx_energy_ionisation	0.009661096
wt_num_PIPISTACK_ring_layer1	0.009581442
wt_pct_hotloop	0.009533636
diff_aaindex_alpha_NH_chemical_shifts	0.009358217
diff_aaindex_weights_for_coil_at_the_window_position	0.009356889
wt_pct_ss_s	0.009251928
diff_aaindex_pK_N	0.009224498
wt_PSSM_score_aver	0.008755336
mut_PSSM_score_aver	0.008613576
wt_num_hd_cluster_protlego_layer1	0.008600293
diff_aaindex_average_relative_fractional_occurrence	0.008402331
layer1_wt_num_pharm_c_hb_donors	0.008194519
diff_aaindex_residues _alpha_helices_thermophilic	0.008189471
diff_aaindex_relative_preference_value	0.008126276
diff_foldx_energy_ionisation	0.008118107
diff_aaindex_aa_composition_of_CYT2	0.008101936
layer2_wt_num_pharm_c_hb_donors	0.007867025
wt_aaindex_weights_for_alpha_helix	0.007832048
mut_PSSM_score_b5	0.007732523
wt_foldx_torsional clash	0.00690614
temperature	0.006739545
wt_pct_aa_c_nonpolar	0.006464203
wt_num_VDW_ring_layer2	0.006349765
diff_num_VDW_ring_layer1	0.005468927
