# Supplementary material for: DDGWizard: Integration of feature calculation resources for analysis and prediction of changes in protein thermostability upon point mutations
Source: PLoS Comput Biol. 2025 Dec 1;21(12):e1013783. doi: 10.1371/journal.pcbi.1013783 (PMC12688154; doi:10.1371/journal.pcbi.1013783)
Supplement: S4 Table — (PDF) [file pcbi.1013783.s004.pdf]

S4 Table . Performance comparison of different MLP hyperparamers with identical 20-fold pair-level cross-validation.

Layer number of MLP	Neuron number per layer	Average R^2	Standard deviation of R^2
1	32	0.37	0.07
2	64/32	0.38	0.06
3	128/64/32	0.42	0.07
4	256/128/64/32	0.41	0.06
5	512/256/128/64/32	0.40	0.06
