# Supplementary material for: DDGWizard: Integration of feature calculation resources for analysis and prediction of changes in protein thermostability upon point mutations
Source: PLoS Comput Biol. 2025 Dec 1;21(12):e1013783. doi: 10.1371/journal.pcbi.1013783 (PMC12688154; doi:10.1371/journal.pcbi.1013783)
Supplement: S5 Table — Hyperparameter ranges used for 100 rounds of Bayesian optimization. (PDF) [file pcbi.1013783.s005.pdf]

S5 Table . Used hyperparameter for Bayesian optimization. Hyperparameter ranges used for 100 rounds of Bayesian optimization.

**Hyperparameters**	**Hyperparameter Ranges**	**Optimal Hyperparameters**
n_estimators	10-1,000	917
max_depth	1-10	7
eta	0.01-1	0.99
subsample	0.1-1	0.97
colsample_bytree	0.1-1	0.69
learning_rate	0.001-0.1	0.08
