# Supplementary material for: DDGWizard: Integration of feature calculation resources for analysis and prediction of changes in protein thermostability upon point mutations
Source: PLoS Comput Biol. 2025 Dec 1;21(12):e1013783. doi: 10.1371/journal.pcbi.1013783 (PMC12688154; doi:10.1371/journal.pcbi.1013783)
Supplement: S6 Table — (PDF) [file pcbi.1013783.s006.pdf]

S6 Table . Comparison results of three $\Delta\Delta$G prediction methods evaluated with the identical cross-validation sets on the low-conservation residue data.

**Methods**	**Average $\mathbf{R^2}$**	γ_{all}	$\boldsymbol{p_{all}}$
DDGWizard	0.51	0.72	-
ACDC-NN [23]	0.20	0.46	< 0.001
DDGun3D [29]	0.16	0.45	< 0.001
