# Supplementary material for: DDGWizard: Integration of feature calculation resources for analysis and prediction of changes in protein thermostability upon point mutations
Source: PLoS Comput Biol. 2025 Dec 1;21(12):e1013783. doi: 10.1371/journal.pcbi.1013783 (PMC12688154; doi:10.1371/journal.pcbi.1013783)
Supplement: S7 Table — (PDF) [file pcbi.1013783.s007.pdf]

S7 Table . Comparison results of three ΔΔG prediction methods evaluated with the identical protein-level cross-validation sets.

**Methods**	**Average R^2**	γ_{all}	p_{all}
DDGWizard	0.42	0.64	-
ACDC-NN [23]	0.39	0.60	0.01
DDGun3D [29]	0.37	0.59	< 0.001
