# Supplementary material for: DDGWizard: Integration of feature calculation resources for analysis and prediction of changes in protein thermostability upon point mutations
Source: PLoS Comput Biol. 2025 Dec 1;21(12):e1013783. doi: 10.1371/journal.pcbi.1013783 (PMC12688154; doi:10.1371/journal.pcbi.1013783)
Supplement: S8 Table — (PDF) [file pcbi.1013783.s008.pdf]

S8 Table . Detailed usage of the computational resources.

Computational resources	Usage
AAindex [36]	AAindex database is used to collect physicochemical properties and amino acid substitution matrices. Its recorded physicochemical property values are allocated to the corresponding wild-type and mutant amino acids. The values of recorded amino acid substitution matrices are allocated to the corresponding mutations.
Biopython (v1.81) [37]	Biopython is used to read protein sequence and structure files to calculate the proportions of different amino acids and different amino acid categories. The "SeqIO.parse()" and "PDBParser()" functions were used to parse protein sequences and structures, and the quantities and proportions of different amino acids and amino acid categories were calculated.
Bio3D (v2.4) [38]	Bio3D is used to calculate the NMA-based atomic fluctuation at the mutation site for both wild-type and mutant proteins. The "bio3d" package in R was used, with the "nma()" function employed to process PDB files. The "fluctuations" element of the "nma" object was accessed to obtain the atomic fluctuation values.
DisEMBL (v1.5) [32]	DisEMBL is used to predict the distribution of disorder regions for both wild-type and mutant proteins. The DisEMBL program was run with protein sequences as input and default parameters applied to obtain the predicted sequences of disordered regions.
DSSP [39]	DSSP is used to calculate the distribution of secondary structures for both wild-type and mutant proteins. The DSSP program was run with the PDB file path as input, and default parameters were applied to calculate the secondary structure information for each amino acid.
FoldX (v5.0) [18]	FoldX is used to calculate energy terms for both wild-type and mutant proteins. The FoldX program was run with the protein PDB file path as input, and the "command" parameter was set to "BuildModel".
PROFbval [34]	PROFbval is used to predict the B-factor of mutant amino acids. The PROFbval program was run with the protein sequence and multiple sequence alignment results as input and default parameters applied to obtain the predicted B-factor values.
Protlego (v1.81) [35]	Protlego is used to calculate the distribution information of hydrophobic clusters for both wild-type and mutant proteins. The "get_structures" function from the Protlego library was used to process PDB files, and the "Protlego_Hydrophobic_Cluster()" function was employed to instantiate the analysis object. This provided information on the number, spatial coordinates, and areas of hydrophobic clusters.
PSI-BLAST (v2.13.0+) [41]	PSI-BLAST is used to perform multiple sequence alignments on the UniRef50 database, using the wild-type and mutant protein sequences as input to generate their PSSMs. The run parameters were set with an "evalue" of 0.001, an "inclusion_ethresh" of 0.02, and 3 iterations.
RDKit (v2023.03.3) [40]	RDKit is used to calculate the distribution of atomic pharmacophores for both wild-type and mutant proteins. The "Chem.MolFromPDBFile()" function and the "factory.GetFeaturesForMol()" function from the RDKit library were used to calculate the number and coordinates of different atomic pharmacophores.
Ring (v3.0) [33]	Ring is used to calculate the residue interaction networks for both wild-type and mutant proteins. The Ring software was used with the PDB file as input, and the result text file with the "ringEdges" suffix was generated. The quantities and spatial coordinates of 6 types of interactions- "HBOND", "SSBOND", "IONIC", "VDW", "PICATION", and "PIPISTACK"-were counted and recorded.
SIFT [42]	SIFT is used to predict possible outcomes of amino acid substitutions. The SIFT program was run with the protein sequence and multiple sequence alignment results as input, with default parameters applied. Three types of predicted outcomes-"TOLERATED", "DELETERIOUS", and "NOT SCORED"-were obtained and recorded.
